# Routine Drainage of Colorectal Anastomoses: An Evidence-Based Review of the Current Literature

**DOI:** 10.1155/2017/6253898

**Published:** 2017-10-12

**Authors:** Sameh Hany Emile, Tito M. Abd El-Hamed

**Affiliations:** General Surgery Department, Mansoura Faculty of Medicine, Mansoura, Egypt

## Abstract

**Background:**

The use of prophylactic drainage after colorectal anastomoses has been long debated. This report aimed to review the current literature discussing routine drainage of colorectal anastomoses highlighting two opposite perspectives (prodrainage and antidrainage) to demonstrate the clinical utility of prophylactic drainage and its proper indications.

**Methods:**

An organized literature search was conducted querying electronic databases and Google Scholar. Articles evaluating the role of routine prophylactic drainage after colorectal anastomosis were included and divided into two categories: articles supporting the use of drains (prodrainage) and articles disputing routine drainage (antidrainage).

**Results:**

There were seven systematic reviews and/or meta-analyses, one Cochrane review, one randomized controlled trial, and six prospective or retrospective cohort studies. Six studies supported prophylactic drainage of colorectal anastomoses; the quality of these studies ranged between grade II and IV. Nine studies recommended against the use of prophylactic drainage, six studies were grade I, one was grade II, and two were grade IV.

**Conclusion:**

Since level I evidence studies including well-designed randomized trials and meta-analyses recommended against the use of pelvic drainage as a routine practice after colorectal anastomoses, we conclude no significant impact of routine drainage on the risk of anastomotic leakage after colorectal anastomoses.

## 1. Background

Anastomotic leakage (AL) following colorectal resection is an ominous complication that can lead to prolonged hospital stay, increased cost, morbidity, and perhaps mortality rates. Although an immense number of studies have thoroughly investigated the predictive risk factors and useful tools for the detection and prevention of colorectal AL, rates of AL remain higher than the optimal desired.

AL is usually diagnosed on the basis of certain clinical, laboratory, and radiological parameters. Clinical features of AL include gross discharge of enteric contents either through the abdominal incision or the pelvic drain, with or without associated septic manifestations. Elevated leucocytic count, C-reactive protein [1], and fluid cytokine levels [2] can be markers for AL; nevertheless, their clinical utility remains questionable. Pelvic ultrasonography, computed tomography scan, and water-soluble contrast studies are the commonly used radiologic modalities for detection of AL [3].

The use of abdominal/pelvic drains after colorectal anastomosis has been debated for a long time and the debate seems to continue. Although several studies, including meta-analyses and randomized trials, concluded no clinical benefit of drainage after colorectal anastomosis, many surgeons worldwide opt to use drains especially with low colorectal or coloanal anastomoses. This review is illustrating two opposite perspectives; the prodrainage and the antidrainage, emphasizing the available evidence supporting each perspective.

## 2. Search Strategy

An organized literature search was conducted querying electronic databases including PubMed/Medline, SCOPUS, and Google Scholar. The following keywords were used in the search process: “Colorectal,” “Colon^∗^,” “Rectal,” “Anastomosis,” “Anastomoses,” “Leakage,” “Leak,” “dehiscence,” “Resection,” “Drainage,” “Drain^∗^,” “Prophylactic,” and “Outcome.” PubMed function “related articles” was used to search further articles. The reference section of each publication was searched manually for relevant articles. Only English-language papers were reviewed.

Articles included were case series, prospective cohort studies, randomized controlled trials, retrospective studies, systematic reviews, and meta-analyses. We excluded editorials, case reports, letters to the editor, and animal studies. Duplicate reports and conference abstracts were excluded. Articles were systematically screened by title, then by abstract screening as an initial step, and subsequently by full-text screening.

## 3. Prodrainage

Surgeons who routinely use pelvic drains believe that drains have three main functions: prophylactic against accumulation of fluid or blood, hence, prevents formation of pelvic hematoma or abscess; early indicator for AL; and therapeutic role in the conservative management of AL by draining the pelvic collection associated with AL and decreasing the severity of systemic sepsis [4].

Peeters and colleagues [5] supported the prophylactic role of drains by reporting a significantly lower rate of AL in drained than nondrained patients (9.6% versus 23.5%) after total mesorectal excision (TME) for rectal cancer located 15 cm or less from the anal verge. Restoration of the bowel continuity was achieved with side to end or colonic pouch anastomosis and diverting stoma was performed in 56.6% of patients, and around 8% of patients with stoma developed AL, significantly less than patients without protective stoma (16%) (*p* < 0.0001). Further analysis revealed that nondrained patients had a relative risk of two and half times more than drained patients for developing AL.

Qu et al. [6] conducted a meta-analysis of the risk factors for AL after laparoscopic anterior resection and found the use of pelvic drains protective against the development of AL (odds ratio [OR] = 0.43, *p* = 0.04). The authors explained this finding that the formation of presacral hematoma or seroma after TME can be a good medium for bacterial infection that may compromise the integrity of the anastomosis. The overall rate of AL was 6.3% (5.3% in drain group versus 23.5% in nondrain group) across the studies. Even if drains did not fully prevent the onset of AL, they still managed to decrease the rates of reoperation for AL [5]. On the other hand, three studies of the meta-analysis used transanal drainage which had no significant impact on the incidence of AL. An important predictor for AL was the level of anastomosis as the incidence of AL was 19.1% for anastomoses within 5 cm from the anal verge compared to 2.3% for anastomoses > 5 cm from the anal verge. Of the 14 trials included in the meta-analysis, eight used double-stapling technique for creation of the anastomosis with rates of AL ranging from 2.6–12.3%. Four trials including 2345 patients used protective stoma in 22.5% of patients and concluded no significant effect of diverting stoma on the rate of AL.

Two studies found pelvic drainage associated with lower rates of AL after laparoscopic anterior resection using the double-stapling technique; however, no statistical significance for the prophylactic role of drainage was obtained. Kawada et al. [7] published a retrospective series of 154 patients with rectal cancer within 10 cm of the anal verge who underwent low anterior resection without diverting stoma. The authors reported AL in 10.8% of drained patients versus 20.8% of nondrained patients (*p* = 0.18). Similarly, transanal drainage which was used in 83% of patients did not reduce the incidence of AL in a significant manner.

Akiyoshi et al. [8] studied 336 patients with rectal carcinoma who underwent low anterior resection with double-stapling technique anastomosis and found drained patients to have lower incidence of AL than nondrained patients (2.6% versus 6.3%) (*p* = 0.11). Transanal tube drainage was used in 63.4% of patients who had comparable AL rates to those without transanal drainage. Protective stoma was employed in 18.4% of patients with no significant difference in the incidence of AL compared to patients without covering stoma.

A meta-analysis [9] of eight trials examined the clinical utility of drainage of extraperitoneal colorectal anastomosis after rectal resection and TME. Two studies employed stapled anastomosis, whereas six employed either hand-sewn or stapled anastomosis. Five studies used covering stoma in select patients whereas three trails did not report the use of diverting stoma in any patient. The meta-analysis demonstrated lower incidence of extraperitoneal colorectal AL in drained patients than nondrained patients (OR = 0.51; 95% CI: 0.36–0.73). Furthermore, drained patients had a significantly lower reintervention rate than patients without drainage (OR = 0.29; 95% CI: 0.18–0.46). It is worthy to mention that subgroup analysis of the three randomized trials included in the meta-analysis found no significant difference between drained and nondrained patients in terms of AL rates.

Drains were once described as the eye watching on the anastomosis [10]; this was asserted by Tsujinaka et al. [11] who studied 196 patients who underwent low anterior resection of rectal cancer with stapled anastomosis in 88% of patients and hand-sewn anastomosis in 12%, both anastomotic techniques had comparable incidence of AL. The authors found pelvic drains to have acceptable sensitivity in detecting AL which occurred in 10.7% of patients overall. Protective ileostomy was used in 23.5% of patients and had no significant impact on the rate of AL. Changes in drain contents suggesting AL was observed in 15 (71.4%) of 21 patients. The study came to a conclusion that “pelvic drainage may act as an early detector of AL and reduce the need for reoperation in selected patients undergoing rectal cancer surgery”.

In addition to direct visual monitoring of the fluids coming out through the drain, several biomarkers [12] such as interleukins, tumor necrosis factor, matrix metalloproteinase, lactate, glycerol, and pO2 can give an idea about the pathophysiologic changes at the anastomosis which may help in the early detection of AL before it is clinically manifested.

Moreover, drains can be considered a component of the management plan for colorectal AL. Should AL supervenes with ongoing collection of enteric materials in the pelvis, adequate drainage is crucial to prevent formation or to drain an already formed pelvic collection or abscess [4]. Drainage is conducted either by the drain inserted during the initial surgery provided that it is properly placed by ultrasound or CT-guided drainage [13]. It has been reported that conservative management of nonsymptomatic AL after anterior resection with drainage only can have a success rate of 48% [11]. Summary of the studies that found drainage after colorectal anastomosis to be of clinical benefit is displayed in Table 1.

## 4. Antidrainage

Surgeons who dispute the use of drains after colorectal anastomosis believe that drains do not only fail the three presumed functions: prophylaxis, alarming, and treatment, but they can also be an independent risk factor for AL and other serious complications.

Five meta-analyses concluded that drains do not reduce the incidence of AL after colorectal procedures; on the contrary, they can induce more harm than benefit. Urbach et al. [14] conducted a meta-analysis of four randomized trials, one of the trials involved colonic anastomoses only whereas the other three trials employed colorectal or coloanal anastomoses in 29–100% of patients. Stapled anastomosis was performed in 11–27% of patients included in the trials. It was found that the drained group has higher rates of clinical leak (OR = 1.5), wound infection (OR = 1.7), and mortality (OR = 1.4) than the nondrained group. Overall, 20 (8.9%) of 223 drained patients developed AL versus 12 (6.4%) of 188 nondrained patients. However, as the authors acknowledge, the power of the analysis was too low to detect significant differences between the two groups. Additionally, the authors reported low sensitivity (5%) of drains in the early detection of AL as only one of 20 drains contained pus or enteric content at the time of diagnosis of leakage.

In the second meta-analysis, Petrowsky and colleagues [15] examined the value of prophylactic drainage in gastrointestinal surgery in general and found level Ia evidence that drains do not reduce complications after colonic or rectal resection with primary anastomosis discouraging the use of prophylactic drainage in these conditions. The meta-analysis included eight trials with different levels of anastomosis: two trials involved colocolic anastomoses only; three comprised colorectal or coloanal anastomoses only; and three involved either colocolic, colorectal, or coloanal anastomoses. The overall rate of AL in drained patients was 4.2% (30 of 717 patients) versus 2.4% (16 of 673 patients) in nondrained patients. However, the value of routine drainage was not clinically established in any of the trials regardless the level of anastomosis.

The third meta-analysis by Karliczek et al. [16] reviewed the outcomes of 1140 patients with elective coloanal anastomoses included in six randomized trials and found comparable rates of clinical AL (2% versus 1%), radiologic AL (3% versus 4%), wound infection (5% versus 5%), reintervention (6% versus 5%), and mortality (3% versus 4%) between drained and nondrained patients, respectively. This meta-analysis also comprised different levels of anastomoses (intraperitoneal in one study, extraperitoneal in another study, and both in four trials) with different indications for surgery, such heterogeneity among the studies that can make the outcome of the meta-analysis less reliable.

The fourth meta-analysis [17] analyzed the outcomes of 11 randomized controlled trials that compared routine use of drainage to nondrainage regimes after colorectal anastomosis. AL developed in 67 (7.1%) of 939 drained patients versus 50 (5.7%) of 864 nondrained patients. The drain group had a relative risk of overall AL equal to 1.14 (*p* = 047), clinical AL equal to 1.39 (*p* = 0.24), radiologic AL equal to 0.92 (*p* = 0.74), wound infection equal to 1.19 (*p* = 0.34), and mortality equal to 0.94 (*p* = 0.81). Zhang and colleagues stated that routine prophylactic drainage in colorectal anastomosis does not reduce AL or other postoperative complications. Akin to its predecessors, this meta-analysis included different levels of anastomosis (extraperitoneal in two trails, intraperitoneal in four trials, and either technique in five trails). Also, the method of anastomosis was quite heterogeneous as four studies applied stapled anastomosis only while the remaining trials used either stapled or hand-sewn anastomosis.

In the most recent meta-analysis, Menahem and colleagues [18] analyzed three randomized trials involving 660 patients with extraperitoneal anastomosis after rectal resection. Two trials employed either hand-sewn or stapled anastomosis, and one trial employed stapled anastomosis only. There were no significant differences between drained and nondrained patients regarding AL (14.8% versus 16.7%) and mortality (0.7% versus 1.9%). On the other hand, patients in the drain group had significantly higher incidence of small bowel obstruction (18.7% versus 12.6%). The authors concluded that pelvic drainage had no effect on the incidence of AL and mortality after extraperitoneal colorectal anastomosis.

A Cochrane review [19] of randomized and nonrandomized trials found no statistically significant difference in clinical AL of patients treated with routine drainage after elective colorectal anastomosis compared to no drainage (1.7% versus 1.2%) with risk ratio: 1.40, 95% CI: 0.45 to 4.40. Similarly, no significant differences in mortality, reintervention, radiological AL, and wound infection rates were observed between both groups. The significant heterogeneity in the level and method of anastomosis and the type of drains used in the studies include to this Cochrane review is a chief limitation that may prevent concluding the actual clinical benefit of drainage of colorectal and coloanal anastomosis after rectal resection in particular.

A large prospective trial [20] on 978 patients with rectal cancer within 15 cm of the anal verge who underwent elective anterior resection discouraged the use of irrigation suction drains as they were associated with a high incidence of AL (OR = 9.13; 95% CI: 1.16–71.76), recommending to use other types of drains when required in difficult operations to prevent formation of hematoma. The authors used different techniques of anastomosis including hand-sewn, single and double stapled, and J pouch; however, no significant difference in the incidence of AL was observed among these techniques. On the other hand, the level of anastomosis was a significant predictor for AL as the OR for low anastomosis (33% of patients) for developing AL was 2.38 with *p* < 0.05.

The largest randomized controlled trial (GRECCAR 5 trial) [21] included 469 patients with rectal cancer (91% had low rectal cancer < 6 cm of anal verge) who underwent low anterior resection and infraperitoneal hand-sewn (*n* = 217) or stapled (*n* = 252) coloanal anastomosis. Protective stoma was applied in 75% of patients. There was no significant difference in the AL rate between drained and nondrained patients (9.3% versus 8.6%, *p* = 0.78). The method and height of anastomosis and the use of diverting stoma did not have a significant influence on the rate of development of AL. Similarly, the use of prophylactic drainage had no impact on the incidence of AL as the rates of pelvic sepsis (16.1% versus 18.0%, *p* = 0.58), surgical morbidity (18.7% versus 25.3%; *p* = 0.83), and reoperation rate (16.6% versus 21.0%; *p* = 0.22) in drained and nondrained patients were similar. The authors came to a conclusion that pelvic drainage of infraperitoneal anastomosis after resection of rectal cancer did not confer any significant benefit to the patient.

A multivariate analysis [22] of the predictive risk factors for AL after small bowel and colorectal anastomoses found that although drains were used in more than 90% of patients, the use of drains did not confer a significant benefit to the patients as 12.4% of drained patients developed AL compared to 15% of nondrained patients (OR = 1.06, *p* = 0.92). Summary of the studies that concluded no clinical benefit of routine drainage after colorectal anastomosis is illustrated in Table 2.

## 5. Analysis of AL in Drained versus Nondrained Patients

Owing to the significant heterogeneity of the studies included, a quantitative analysis of the outcome of drainage of colorectal anastomosis was not possible. Nonetheless, on collective analysis of the studies, the rates of AL in drained and nondrained patients were compared with a median of 7.2% (1.7–14.8) in the drain group versus 6.4% (1–23.5) in the nondrain group. Protective stoma was performed in a median of 22.5% of patients, ranging from zero to 75% (Table 3).

## 6. Complications and Drawbacks of Drains

Drains can be obstructed or clogged by blood clots or tissue debris or displaced away from the vicinity of the anastomosis. Therefore, a nonfunctioning drain is not only of no value, but it may give a false sense of security to the surgeons as they observe no significant drainage in the tube while seroma or hematoma is building up slowly inside the pelvis.

Moreover, although drainage is thought to guard against AL, it can become an independent risk factor for AL. According to Yeh et al. [20], irrigation suction drains are associated with higher incidence of AL compared to Silastic Penrose or Jackson-Pratt drains. A univariate analysis [23] of the risk factors for AL in 1576 patients with colorectal anastomosis identified the use of drains as a significant predictor of AL as 8.5% of drained patients developed AL compared to 5.1% of nondrained patients. Nevertheless, the nonrandomized nature of both trials casts the risk of bias on their findings which prevents reaching interim conclusions.

Complications of drains are underreported in the literature, and detailed description of drain-related complications is seldom reported except in the form of case studies. The drain-related morbidities include pain at the site of drain, skin maceration and excoriation, wound infection, bleeding, intestinal injury in around 0.5% of patients, and herniation of the omentum in up to 1% of patients [4, 11, 24, 25].

Drains can be left for long duration when employed for conservative management of AL [11]. Theoretically, the longer the drain stays, the higher is the risk of fistulization, intestinal obstruction or injury, and infection [26]. Factors that can increase the risk of developing drain-related complications include debilitated patients, chronic use of corticosteroids, and large stab incision for drain insertion [27].

## 7. Discussion

Despite the multitude of high-quality studies that discourage the use of prophylactic drainage in colorectal anastomosis, a considerable percentage of surgeons still opts to use drains in colorectal surgery routinely, particularly when the use of drains is left at the discretion of the operating surgeon. Sakr et al. [22] stated that drains were placed in 91% of the patients who underwent small intestinal or colonic anastomoses in their series. Similarly, Hoffmann et al. [24] disclosed that drainage after anterior resection was employed routinely in 97% of the patients recruited in their randomized trial. Interestingly, both studies concluded no tangible benefits nor serious complications of the use of drains after colorectal anastomosis.

It is worthy to note that all of the studies that documented the clinical benefit of routine drainage (prodrainage studies) involved patients who had extraperitoneal colorectal or coloanal anastomoses after rectal resection. The majority of the prodrainage studies used stapled anastomosis technique, and many studies used protective stoma. Some studies applied transanal tube drainage; however, it did not manage to reduce the rate of AL significantly after surgery except in one study [5].

On the other hand, the antidrainage studies were quite heterogeneous including different levels and methods of anastomosis. Only two trials [20, 21] that evaluated the clinical utility of drainage of infraperitoneal colorectal anastomosis after rectal resection found routine drainage to be of no clinical benefit. The remaining articles combined colocolic anastomoses with colorectal and ileorectal anastomoses in the same analysis which might have led to the conclusion of no significant value of drainage since colocolic anastomoses, that do not require drainage on a routine basis, were included in the analysis.

Perhaps, instead of totally avoiding drainage altogether, we should determine when drainage is indicated and which patients need to be drained after colorectal anastomosis. The risk factors for AL have been thoroughly discussed in the literature; these factors can usefully guide the decision of using drains. Drains can be selectively used when significant risk factors for AL such as low pelvic anastomoses, lack of diverting stoma, use of neoadjuvant chemoradiation therapy, emergency surgery, high ASA grade, and chronic liver diseases exist [22, 28–30].

Alternatives to pelvic drainage include the use of intraluminal biodegradable and protective device and transanal drainage (TD). Zhao and colleagues [31] investigated the utility of TD after anterior resection for rectal cancer and found TD to reduce the incidence of AL and anastomosis-related complications from 11.7% in the control group to 2.5% in the TD group. However, these findings did not attain statistical significance owing to the small number of patients included in the study. Similarly, two large studies in the present review [7, 8] evaluated the use of TD in conjunction with pelvic drainage of colorectal anastomosis after low anterior resection and found neither types of drainage reduce the incidence of AL in a significant manner. On the other hand, a recent meta-analysis [32] including 909 patients reported TD to have significantly lower rates of AL (OR = 0.30; *p* = 0.0001) and reoperation (OR = 0.18; *p* = 0.0002) after anterior resection than the control group.

## 8. Summary and Conclusions

Since level I evidence including well-designed randomized trials and meta-analyses recommended against the use of pelvic drainage as a routine practice after colorectal anastomosis, we can conclude that routine drainage has no significant impact on the rate of colorectal AL but may have a selective utility when the operative field is not dry in order to decrease the need for surgical drainage of fluid collection or abscess, even when AL is not present.

In order to overcome the drawbacks and complications of drains, clear guidelines about when and how to employ drainage after colorectal anastomosis should be designed and implemented. Alternatives to pelvic drainage exist; however, the paucity of reliable evidence on their efficacy and safety neceesiates more prospective studies.

## Figures and Tables

**Table 1:** Summary of the studies that favored routine drainage after colorectal anastomosis.

Studies of prodrainage	Type of study	Level of evidence^†^	Level of anastomosis	Method of anastomosis	Use of diverting stoma
Peeters et al. 2005 [5]	Retrospective	VI	Colorectal	Side to end or colonic pouch anastomosis	56.6%
Qu et al. 2015 [6]	Systematic review and meta-analysis	IIa	Colorectal	Double stapled in 8 trials and handsewn or stapled in 6 trials	22.5% (4 trials)
Kawada et al. 2014 [7]	Retrospective	VI	Colorectal	Double stapled	0
Akiyoshi et al. 2011 [8]	Prospective cohort	IIb	Colorectal	Double stapled	18.5%
Rondelli et al. 2014 [9]	Systematic review and meta-analysis	IIa	Colorectal	Stapled in 2 trials and handsewn or stapled in 8 trials	43.5% (five trials)
Tsujinaka et al. 2008 [11]	Retrospective	VI	Colorectal	Stapled 88% and handsewn 12%	23.5%

† indicates levels of evidence proposed by the Oxford Centre for Evidence-Based Medicine (Meakins JL. Innovations in surgery: the rules of evidence. Am J Surg. 2002; 183: 399–405).

**Table 2:** Summary of the studies that discouraged routine drainage after colorectal anastomosis.

Studies of antidrainage	Type of study	Level of evidence^†^	Level of anastomosis	Method of anastomosis	Use of diverting stoma
Urbach et al. 1999 [14]	Meta-analysis of randomized trials	Ia	Colocolic in 1 trial (52%) and colorectal or coloanal in 3 trials (48%)	Stapled in 11–27% and handsewn in 73–89%	NA
Petrowsky et al. 2004 [15]	Systematic review and meta-analysis	Ia	Colocolic in 2 trials, colorectal in 3 trials, and colocolic or colorectal in 3 trials	NA	NA
Karliczek et al. 2006 [16]	Meta-analysis of randomized trials	Ia	Intraperitoneal in 1 trial, extraperitoneal in 1 trial, and intra- or extraperitoneal in 4 trials	NA	NA
Zhang et al. 2016 [17]	Meta-analysis of randomized trials	Ia	Intraperitoneal in 4 trials, extraperitoneal in 2 trials, and intra-or extraperitoneal in 5 trials	Stapled in 4 trials, and handsewn or stapled in 7 trials	NA
Menahem et al. [18]	Meta-analysis of randomized trials	Ia	Extraperitoneal	Stapled in 1 trial and stapled or handsewn in 2 trials	NA
Rolph et al. 2004 [19]	Cochrane review	IIa	Colocolic in 2 trials and colorectal or coloanal in 1 trial	Stapled in 46.7% and handsewn in 53.3%	NA
Yeh et al. 2005 [20]	Retrospective	VI	Colorectal	J pouch (16%), single stapled (3.5%), double stapled (70.7%), and handsewn (6.8%)	10%
Denost et al. 2016 [21]	Randomized controlled trial	Ia	Infraperitoneal colorectal	Handsewn 46.3% and stapled 53.7%	75%
Boccola et al. 2010 [23]	Retrospective	VI	Colocolic in 46.8%, colorectal in 48%, and ileorectal in 5.2%	Handsewn in 52% and stapled in 48%	6%

NA: not available. † indicates levels of evidence proposed by the Oxford Centre for Evidence-Based Medicine (Meakins JL. Innovations in surgery: the rules of evidence. Am J Surg. 2002; 183: 399–405).

**Table 3:** Rates of AL in drained and nondrained patients in the studies included.

Study	Number	AL in drained group (%)	AL in nondrained group (%)	Use of protective stoma (%)
Peeters et al. [5]	924	9.6	23.5	56.6
Qu et al. [6]	4580	5.3	9.2	22.5
Kawada et al. [7]	154	10.8	20.8	0
Akiyoshi et al. [8]	336	2.6	6.3	18.5
Rondelli et al. [9]	2277	7.2	7.4	43.5
Tsujinaka et al. [11]	196	10.7	NA	23.5
Urbach et al. [14]	414	8.9	6.4	NA
Petrowsky et al. [15]	1390	4.2	2.4	NA
Karliczek et al. [16]	1140	2	1	NA
Zhang et al. [17]	1803	7.1	5.7	NA
Menahem et al. [18]	660	14.8	16.7	NA
Rolph et al. [19]	908	1.7	1.2	NA
Yeh et al. [20]	978	2.8		10
Denost et al. [21]	469	9.3	8.6	75
Boccola et al. [23]	1576	8.5	5.1	6
Median	—	7.2 (1.7–14.8)	6.4 (1–23.5)	22.5 (0–75)

AL: anastomotic leakage. NA: not available.
